# A low-cost naphthaldiimide based organic cathode for rechargeable lithium-ion batteries

**DOI:** 10.3389/fchem.2022.1056244

**Published:** 2022-11-17

**Authors:** Zhuo Wang, Pengchao Zhang, Junpeng Li, Chong Zhang, Jia-Xing Jiang, Menglan Lv, Zhengping Ding, Bin Zhang

**Affiliations:** ^1^ School of Chemistry and Chemical Engineering, Guizhou University, Guiyang, China; ^2^ Key Laboratory for Macromolecular Science of Shaanxi Province, Shaanxi Engineering Laboratory for Advanced Energy Technology, School of Materials Science and Engineering, Shaanxi Normal University, Xi’an, China; ^3^ School of Materials Science and Engineering, Changzhou University, Changzhou, China

**Keywords:** naphthaldiimides, organic cathode, electrochemical properties, capacity, lithium-ion batteries

## Abstract

Recently, the development of cathode materials is becoming an important issue for lithium-ion batteries (LIBs). Compared with inorganic cathodes, the organic cathodes are developing rapidly, ascribing to their distinct merits in light weight, low cost, massive organic resources and high capacity. In this paper, a cost-efficiency naphthaldiimide (NDI) based derivative, 2,7-bis(2-((2-hydroxyethyl) amino) ethyl) benzo[lmn] [3,8] phenanthroline-1,3,6,8(2H, 7H)-tetraone (NDI-NHOH), was used as organic cathode in LIBs. The NDI-NHOH was synthesized easily *via* one-step process, and it showed very high thermal stability. Through mixing NDI-NHOH with acetylene black and polyvinylidene fluoride (weight ratio of 6:3:1) as composite cathode in lithium-metal based LIBs, the NDI-NHOH presented versatile electrochemical properties. From cyclic voltammetry (CV) test, it exhibited two reversible peaks for oxidation and reduction in the first cycle, respectively. Notably, the oxidation and reduction peaks were located at 2.54, 3.22 and 2.14, 2.32 V vs. Li^+^/Li, respectively. By employing NDI-NHOH as cathode, it demonstrated a specific capacity of about 80 mAh g^−1^ in the range of 1.5–3.5 V, where the batteries retained a capacity retention of 50% over 20 cycles. According to the LIBs study, it suggests that the NDI-NHOH-based derivative shows a potentially promising candidate as efficient organic cathode materials for high-performance metal-ions batteries.

## 1 Introduction

Lithium-ion batteries (LIBs) have become the main energy storage and energy supply systems for many portable electronic devices, electric vehicles, and aerospace equipment due to their merits in high energy density, high coulombic efficiency, low self-discharge characteristics and low environmental pollution (Kim et al., 2019)^1^. The energy storage of transition metal-based inorganic electrode materials in LIBs is limited by the change of transition metal oxidation state and the irreversible capacity loss caused by the release of lattice oxygen. These negative factors essentially impair the practical application of inorganic materials (Yuan et al., 2022)^2^. LIBs are mainly composed of four parts: cathode, anode, electrolyte, and separator. Among them, the cathode materials are the core component of the whole system, including inorganic and organic cathode materials, whose characteristics have significantly impacted the battery performance. Currently, the commercial cathodes are inorganic materials, mainly dividing into transition metal oxides, phosphate and cobaltate compounds, such as olivine (LiFePO_4_), lithium cobaltate (LiCoO_2_) and lithium manganate (LiMnO_2_) (Mahmud et al., 2022)^3^. However, ascribing to the small theoretical capacity, high cost and limited resources, these inorganic materials urgently need to be replaced by new electrode materials (Lyu et al., 2021)^4^.

Due to their high specific capacity, flexible design, and environmental friendliness, organic materials have become a research hotspot in the area of rechargeable batteries, in contrast to the major concerns of high recycling costs and environmental contamination that inorganic materials may cause (Liu et al., 2022)^5^. Most organic materials used in rechargeable batteries are carbonyl compounds, in which the carbonyl (C=O) has high redox activity. For instance, a novel anthraquinone derivative with multiply symmetrical hydrophilic hydroxyl groups (1,3,5,7-THAQ), was reported by Wang’s group, where the hydrophilic hydroxyl groups effectively improved water solubility and provided the high redox activity (Wang et al., 2022)^6^. During the discharge process of the battery, the carbonyl group is reduced to form an oxygen anion, which is coordinated by the cation in the electrolyte (Häupler et al., 2015)^7^. Therefore, exploring a compound with a larger number of carbonyl groups and a smaller molecular weight is a strategy to gain electrode materials with high specific capacity (Liang et al., 2013)^8^. Notably, the comparatively weak intermolecular interactions of organic materials create a natural area for tolerating different metal ions through cycling. (Xie and Zhang, 2019)^9^. However, the conductivity of organic materials is poor, which is an important effect on LIBs (Xie and Zhang, 2016)^10^. Also, there are some problems, such as persistent dissolution, redeposition, and persistent battery capacity reduction, caused by the high solubility of organic materials. Zheng et al. (2021) synthesized a conjugated polymer of polyphenyl-1,3,5-(pyrene-4,5,9,10-tetraone) (PPh-PTO) by a coupling reaction, aiming to inhibit the solubility of polymer (Zheng et al., 2021)^11^. These weaknesses in organic electrodes prevent the development of practical applications of organic materials in LIBs (Liu et al., 2022)^12^.

Naphthaldiimides (NDI) is a class of planar and aromatic n-type organic compounds with high redox activity (Kuang et al., 2019)^13^. The NDI-based molecules are commonly used in organic electronic devices, such as organic photovoltaic cells (Liu et al., 2020)^14^, organic field-effect transistors (Park et al., 2022)^15^ and sensors (Ali et al., 2022)^16^ in previous studies. In addition to its small molecular weight and easy modification, NDI also has excellent electrochemical properties. In recent years, NDI has attracted much attention ascribing to its remarkable specific capacity and outstanding electrochemical exchange performance, especially in the research field of electrochemical energy storage (Zhou and Han, 2021).^17^ Biradar’s team designed and synthesized the NDI-1DP/CP and NDI-2DP/CP by introducing dopamine (DP)-functionalized derivatives at the imide position of NDI. The excellent stability was demonstrated by NDI-2DP/CP up to 10,000 charge/discharge cycles, and the retention of initial capacitance was almost 96% in the three-electrode configuration setup (Biradar et al., 2021)^18^. Gu et al. constructed 3D polyimide frameworks by one-step polymerization and used them as cathodes for LIBs. The 3D polyimide electrode exhibited 96% capacity retention after 100 cycles at 50 mA g^−1^ and 93% capacity retention after 3,000 cycles at 1,000 mA g^−1^. This electrode also demonstrated excellent long-term stability and reversibility in cycling (Gu et al., 2021).^19^ Generally, NDI derivatives are realized by high electron affinity, which can be effectively tuned by different donor substitutions (Masimukku et al., 2022).^20^ The two-dimensional NDI-based polymer (2D-PAI@CNT) reported by Wang showed excellent performance due to its unique π-conjugated structural unit, which fully exhibited the redox characteristics of the NDI unit. These doped carbon nanotubes show an obvious porous structure with carbonyl groups as active groups, and the utilization rate can reach more than 83%, ensuring the efficient diffusion of ions in 2D molecular channels (Wang et al., 2019)^21^. Therefore, the synthesis strategies of NDI derivatives are mainly to functionalize the structure or expand their conjugated systems to increase redox-active centers. In this way, the performance of cycling stability, solubility, and conductivity of its derivatives can be regulated effectively (Chen et al., 2017)^22^. For example, Wu et al. (2021) prepared a 3D π-conjugated covalent triazine core framework (Azo-CTF) with triazine as the electron-rich center and azo unit as the redox active linkers (Wu et al., 2021)^23^. This demonstrates that the strategy of synchronously orderly introducing electron withdrawing units and redox active units into the conjugated polymer backbone is feasible and effective. In addition, NDI has also appeared in the research of aqueous ion batteries. Since the repeated co-intercalation/extraction of hydrated H^+^/Zn^2+^ can lead to the volume expansion of the material (Zhang et al., 2022)^24^, some researchers have suppressed H^+^ intercalation by introducing NDI to control the depth of discharge (Na et al., 2020)^25^.

In this study, we focused on exploring the electrochemical properties of an NDI- derived organic cathode material. It is wildly noted that, by modifying the conjugated structure, the cycle stability of the battery can be optimized, multi-electron reactive groups can be constructed, redox active sites can be increased, and the proportion of inactive groups can be reduced. So, the flexible structure and easily tunable performance parameters of NDI make it possible to optimize the cycling stability of batteries. Here, we synthesized an NDI derivative of 2,7-bis (2-((2-hydroxyethyl) amino) ethyl) benzo [lmn] [3,8] phenanthroline-1,3,6,8(2H, 7H)-tetraone (NDI-NHOH) *via* one-step process, and analyzed the relationship between molecular structure and electrochemical performance when NDI-NHOH was used as a cathode material in LIBs. For solving the poor conductivity of the material, NDI-NHOH was mixed with acetylene black and polyvinylidene fluoride (weight ratio of 6:3:1) as the composite cathode for lithium metal-based LIBs. After testing, it shows a first discharge capacity of 78.1 mAh g^−1^ at 0.1 C, and still maintains 68.0% after 20 cycles. The physical properties, including infrared absorption spectroscopy (IR), X-ray diffraction (XRD), scanning electron microscope (SEM), energy dispersive spectroscopy (EDS) and X-ray photoelectron spectroscopy (XPS), were also carried out. The characterization results show that the electrochemical performance is effectively improved when NDI-NHOH is used as a composite cathode. Through this study, it can further expand the research interests on the mechanism of NDI composite electrodes and provide helpful experience for the design and synthesis of similar new materials. At the same time, we hope to provide potential options for organic electrode materials in LIBs.

## 2 Experimental

### 2.1 Synthesis

The target organic small molecule NDI-NHOH was synthesized according to the published paper, which was prepared feasibly *via* the one-step process (Sissi et al., 2007)^26^. To obtain the NDI-NHOH, the reactants of 1,4,5,8-naphthalenetetracarboxylic dianhydride and N-(2-hydroxyethyl) ethylenediamine were refluxed in DMF for 8 h.

### 2.2 Batteries fabrication and performance measurement

Electrochemical performance tests were completed in an Ar-fulfilled glove box using CR2025-type coin cells. Using Li metal as the counter electrode, Celgard 2,500 as the separator, and 1 M LiTFSI in EC-DMC (1:1 v/v) as the electrolyte, the coin cells were built. Active material (organic material), acetylene black, and polyvinylidene fluoride (PVDF) were mixed with N-methyl-2-pyrrolidone (NMP) in a weight ratio of 6:3:1 to produce the working electrode. The slurry was then applied on Al foil and dried for 12 h at 120°C in a vacuum oven. For the cathode, a 12-mm-diameter disc of the produced electrode with an areal coating density of around 0.80 mg/cm2 was fashioned. The battery testing equipment (Newware CT-4008, P. R. China) was used to conduct galvanostatic charge-discharge experiments in the potential range of 1.5–3.5 V (1 C = 350 mAh g^−1^). Cyclic voltammograms (CV) were recorded from the electrochemical workstation of the CH660E model (CH660E, CH Instruments, Chenhua Co., Ltd, Shanghai) at room temperature. IR measurements are performed on Bruker Tensor 27 spectrometer (Bruker Optics, Inc., Billerica, MA). XRD measurements are performed on a D/Max-3c multifunctional X-ray diffractometer at angles of 0°–80° (Dandong Haoyuan Instrument CO., Ltd, China). At the same time, the micro-morphological characterizations are carried out on the SEM of the model SU 8020 (Hitachi, Japan). EDS is done on the EMAX evolution X-Max 80/EX-270 (HORIBA, Japan). XPS test is finished on model Escalab Xi+ (Thermo Fisher Scientific, Waltham, United States).

## 3 Results and discussion

### 3.1 Scanning electron microscope, energy dispersive spectroscopy and x-ray photoelectron spectroscopy analysis

**FIGURE 1:**
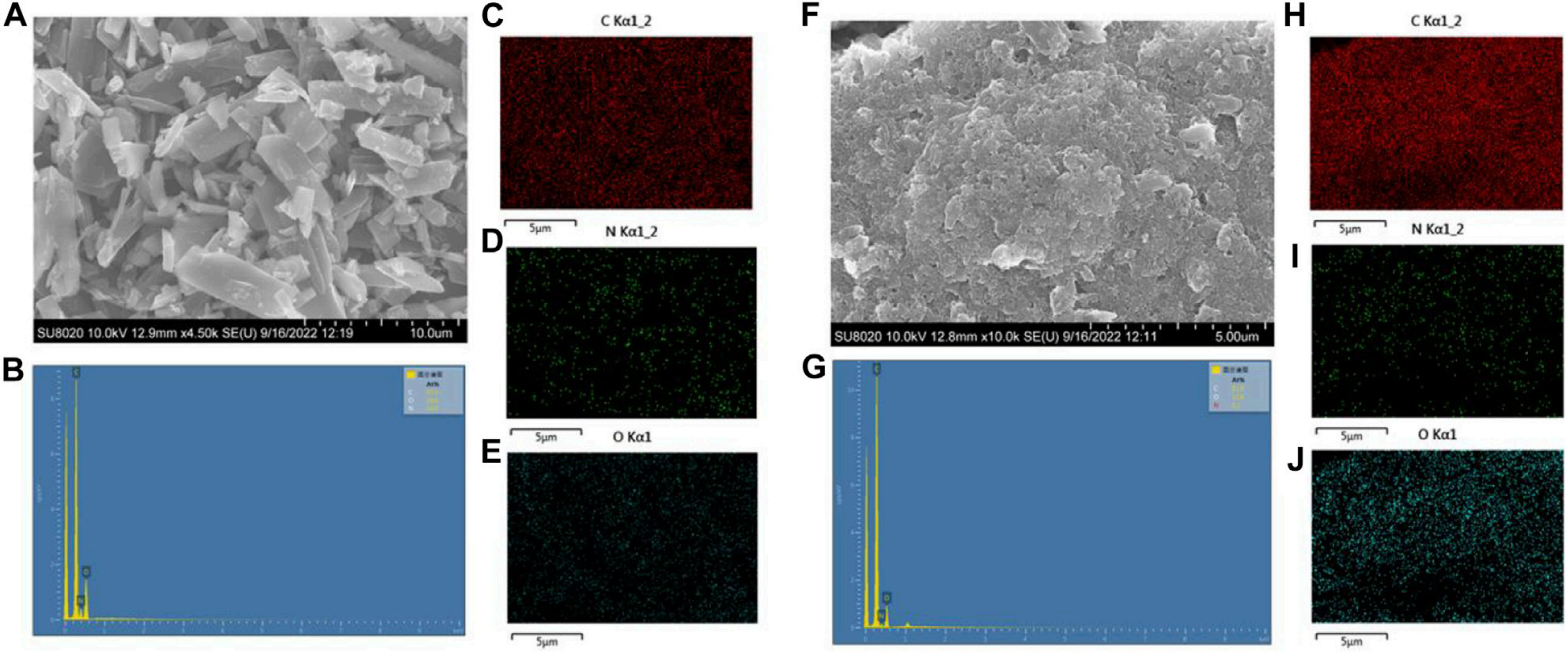
**(A)** SEM images of NDI-NHOH sample, **(B)** element content spectrum of NDI-NHOH sample, **(C–E)** elemental C, N, O mapping images of raw NDI-NHOH sample, respectively; **(F)** SEM images of NDI-NHOH based electrode, **(G)** element content spectrum of NDI-NHOH based electrode, **(H–J)** elemental C, N, O mapping image of NDI-NHOH based electrode, respectively.

The SEM was characterized to investigate the surface morphology of the organic cathode. As shown in Figures 1A,F, the powder sample of NDI-NHOH is mainly elongated structures with some small particles whose molecular stacking is disordered, and the arrangement is not uniform. While, the particles observed by the mixed electrode have been tightly agglomerated and become uniform and orderly because of the addition of acetylene black and polyvinylidene fluoride. At the same time, it can be observed that acetylene black is successfully attached to the NDI-NHOH, which has become a relatively small-sized material with a large surface area. The steric order and the smaller size of the materials can provide a larger specific surface, which can effectively improve the ion transport between the electrode and the electrolyte (Xu, et al., 2008)^27^. In addition, it is observed in the SEM image that acetylene black is well covered on NDI-NHOH after adequate mixing. This is also confirmed by the EDS images (Figures 1B–E,G–J) that the NDI-NHOH is mixed well in the composite cathode materials when acetylene black exists as a conductive moiety. This performance implies that the preparatory purpose of increasing the conductivity of the electrode by mixing acetylene black has been achieved (Merzouki and Haddaoui, 2012)^28^.

**FIGURE 2:**
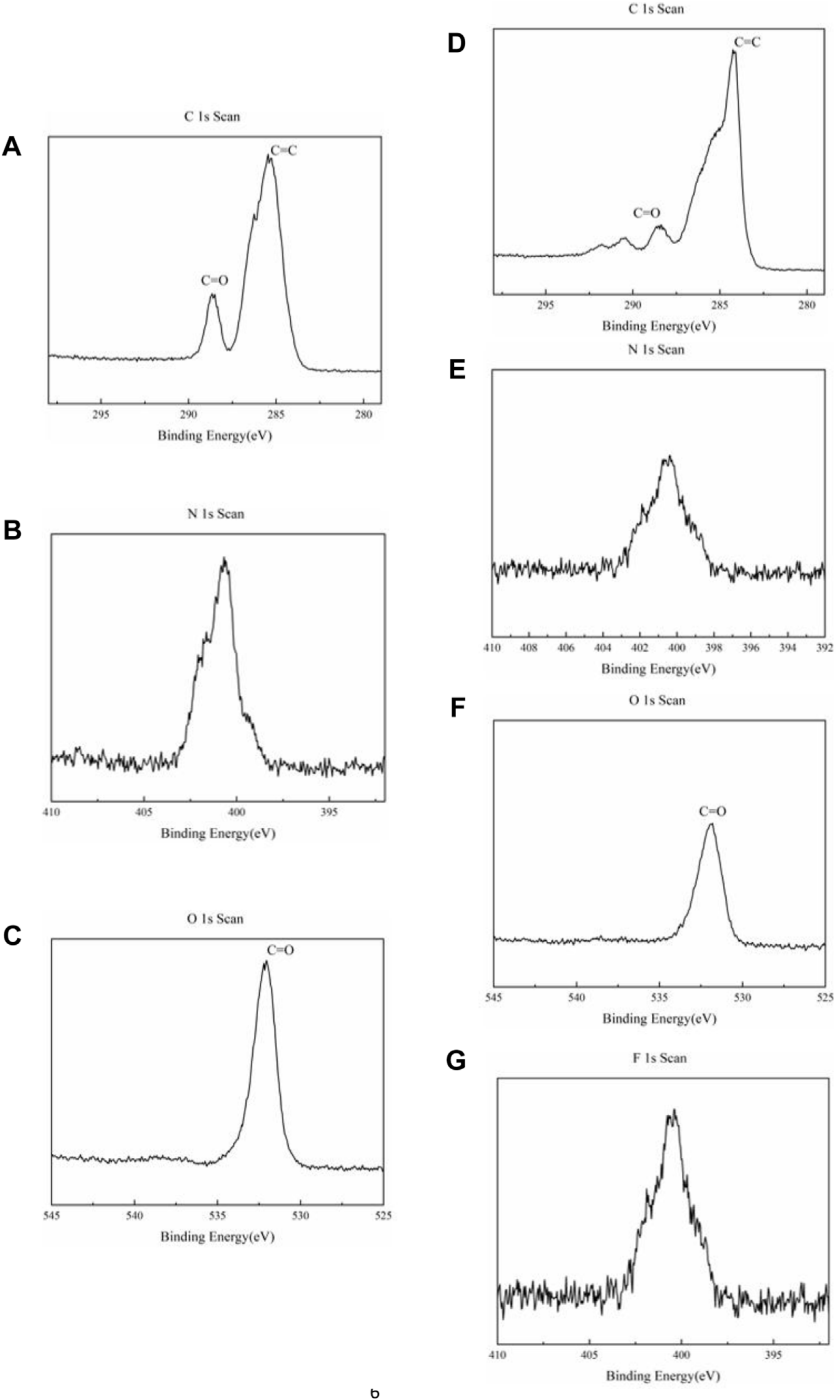
XPS spectra of NDI-NHOH sample: **(A)** C element, **(B)** N element and **(C)** O element; XPS spectra of NDI-NHOH based electrode: **(D)** C element, **(E)** N element, **(F)** O element and **(G)** F element.

The XPS measurement was characterized to study the elementary composition and sample surface further. As shown in Figures 2A–C of raw NDI-NHOH, the spectra of C 1s contain two peaks located at 286.3 and 288.7 eV, respectively, which correspond to the groups of C=C and C=O bonds, respectively. A peak at 532.2 eV can be observed as the O 1s spectrum, corresponding to the C=O group. It can be found that when NDI-NHOH is mixed into an electrode sheet, the structure of C=O decreases and C=C increases due to the addition of acetylene black (Figures 2D–G). The peak of N element is observed at about 400 eV in the spectrum of N 1s, but the peak of N element is reduced due to the addition of acetylene black and polymer binder. In addition, a strong peak of F element was also detected at 688 eV in the electrode due to the addition of polyvinylidene fluoride as the binder.

### 3.2 X-ray diffraction and IR analysis

**FIGURE 3:**
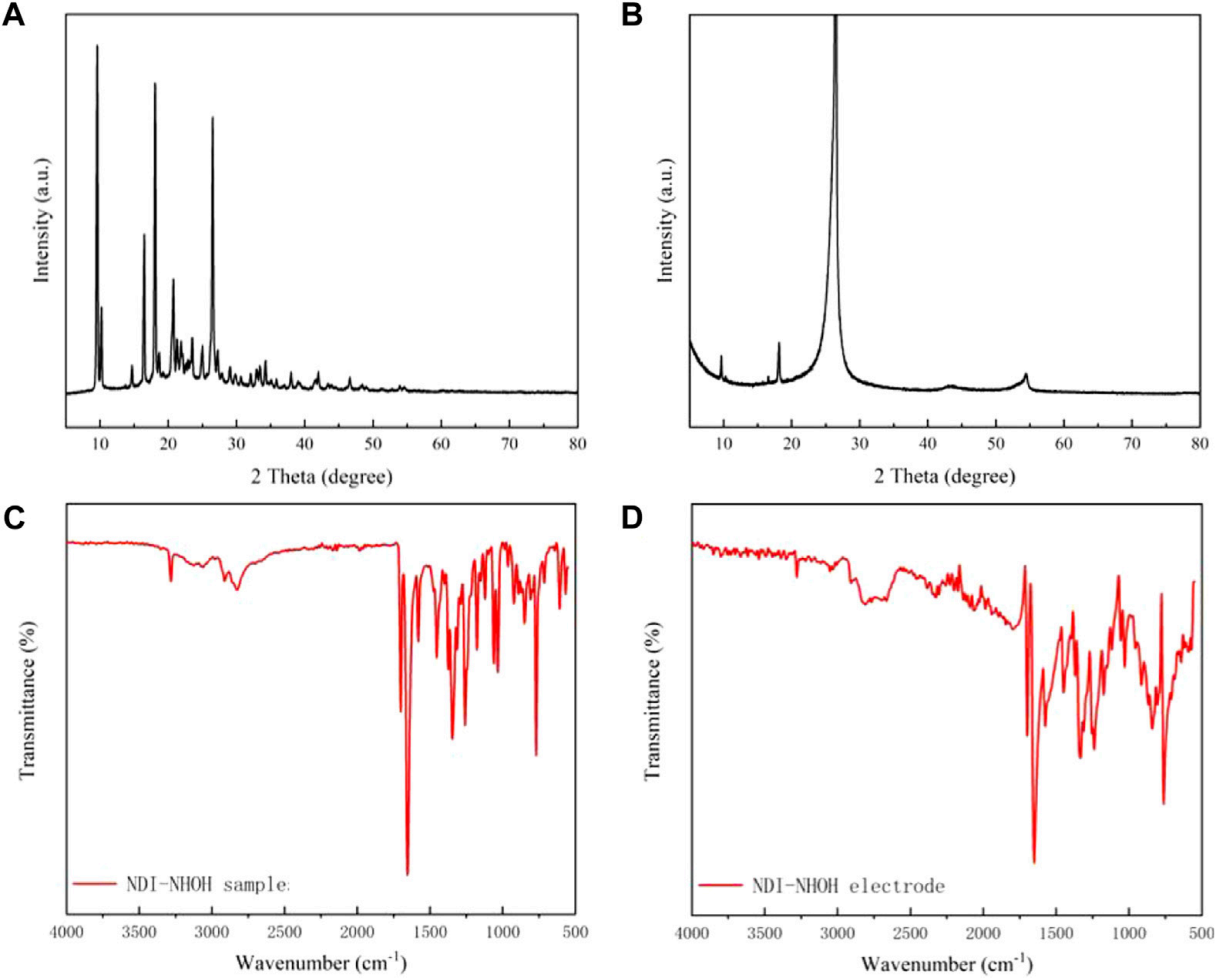
XRD curves of **(A)** NDI-NHOH sample, and **(B)** NDI-NHOH based electrode; IR curves of **(C)** NDI-NHOH sample, and **(D)** NDI-NHOH based electrode.

The XRD was used to measure the raw NDI-NHOH and composite materials to test the stacking properties of molecule and cathode materials. In this test, the scan angle is in the range of 0–80°. It can be seen from Figure 3A that six diffraction peaks are observed in the NDI-NHOH powder, where the first diffraction peak appears at 9.63°. In addition, the diffraction peaks of NDI-NHOH clearly appeared with different intensities between 9 and 27°. This indicates that the NDI-NHOH has a high degree of crystallization, but the orientation of the crystals is relatively disordered, which is also confirmed by the results observed in Figure 1. From Figure 3B, it can be seen that the NDI-NHOH-based electrode observes a more obvious diffraction peak at 26.5° with a greater intensity, which indicates that under the influence of the high adhesion of PVDF and conductive acetylene black, the crystallinity of the mixed electrode is changed and the materials mixing is better (Wang et al., 2020)^29^.

In order to investigate the molecular composition in the mixed cathode materials, the IR measurement was used to analyze these materials. As shown in Figure 3C, in the NDI-NHOH sample, it has a distinct C-H bending vibration peak at 2,810 cm^−1^ and a C=O stretching vibration at 1,654 cm^−1^. In addition, the NDI-NHOH molecule has a distinct N-H in-plane bending vibration peak at 1,574 cm^−1^ and an O-H out-of-plane bending vibration peak at 761 cm^−1^. In Figure 3D, due to the mixing of NDI-NHOH and acetylene black, the shape of the C=O peak has a certain influence, but there are basically stretching vibrations near 1,654 cm^−1^ in both figures. Notably, due to the introduction of a large amount of acetylene black and polymer binder, it can be found that the absorption and stretching vibration peaks of C-O and C-N change sharply around 1,000 cm^−1^ (Figure 3D). Other peaks, such as N-H and O-H characteristics, are almost unchanged. Overall, the changes in the IR are consistent with changes in the structure of the molecule and the mixing materials (Wei et al., 1994)^30^.

### 3.3 Electrochemical properties

**FIGURE 4:**
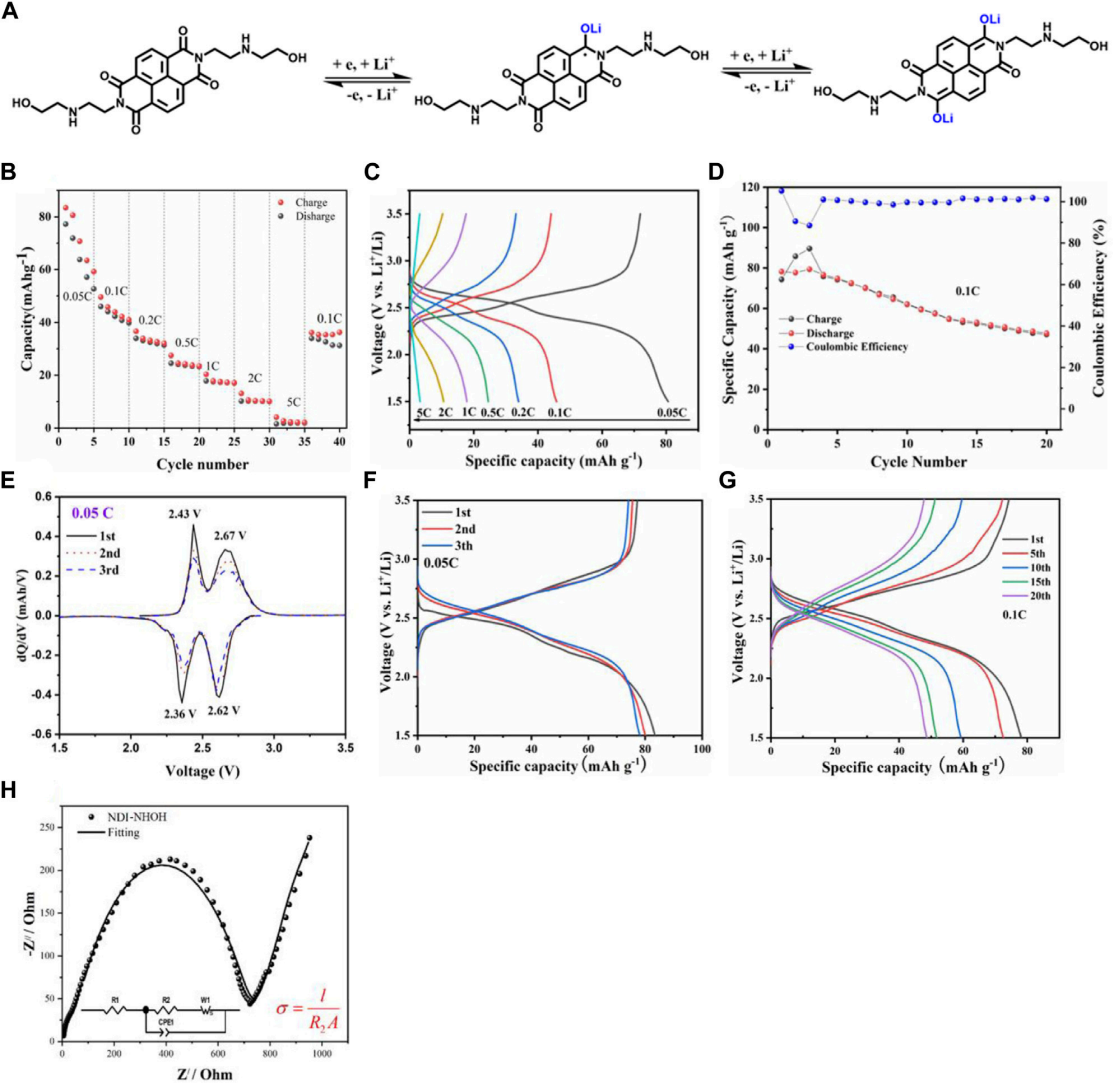
**(A)** The work mechanism of NDI-NHOH electrode; **(B)** rate performance, **(C)** charge-discharge profiles, and **(D)** charge-discharge cycle curves; **(E)** differential capacities (dQ/dV) vs. voltage plots for the initial charge-discharge curves at 0.05 C of NDI-NHOH electrode, **(F)** Galvanostatic discharge and charge curves at 0.05 C and **(G)** Galvanostatic discharge and charge curves at 0.1 C; **(H)** Nyquist plot from EIS measurement.

The work mechanism of NDI-NHOH as cathode electrode in LIBs is present in Figure 4A. It shows that the existed C=O groups in NDI-NHOH can accept the lithium ions easily to form a C-O-Li structure, where the entire molecule can undergo a redox reaction based on two lithium ions. Through the calculation, the theoretical capacity of NDI-NHOH was about 122 mAh g^−1^.

To characterize the battery performance, we tested the charge and discharge process at the different currents from 0.05, 0.1, 0.2, 0.5, 1.0, 2.0–5.0 C, respectively (as shown in Figure 1B). The capacity fluctuations in the first five cycles at 0.05C rate can be attributed to dissolution of organic active materials in electrolytes and incomplete lithiation/delithiation in EC-DMC (Sun et al., 2021 ^31^; Chang et al., 2018 ^32^). The discharge specific capacity of NDI-NHOH can reach 83.42, 45.80, 36.63, 27.49, 20.33 13.10, and 2.7 mAh g^−1^, respectively. From this point, it can be seen that the active material utilization rate of this material is low at high current, and the discharge capacity at 0.5C drops to about 30 mAh g^−1^, which may be due to the nature of the electrolyte itself. It is wildly noted that the ionic electrolyte (1M) has a certain viscosity, and rapid charge and discharge under a high current will reduce the dissociation of ions to a certain extent (MacFarlane et al., 2007)^33^. After 35 cycles, when the current returned to 0.1C immediately, the discharge capacity reached 36.23 mAh g^−1^ at the same time, and there was a slow upward trend. This shows that the recovery of the charging capacity is acceptable. The charge/discharge curves of NDI-NHOH cathodes at different current densities are shown in Figure 1C. The discharge curve at 0.05 C shows the longest second discharge plateau. Although the current density increases inevitably leading to an increase in overpotential, the NDI-NHOH cathode can provide a smooth discharge plateau even at 0.2C. As shown in Figure 1D, the capacity retention rate of NDI-NHOH during long cycling is relatively good at the current of 0.1C, where the first discharge capacity is 78.1 mAh g^−1^, and the capacity after 20 cycles is also 53.1 mAh g^−1^. This is 68.0% of the initial capacity with a capacity decay rate of 1.9% per cycle, which indicates that this cathode shows good cycle stability. It is speculated that the decay of discharge capacity is related to partially irreversible changes in the structure of the material after cycling, such as the reduction of the C=O bonds. It can be seen from the test results in Figure 1D that the coulombic efficiency from the 4th to 13th times is stable at above 99%, and the coulombic efficiency after the 13th time rises and stabilizes above 100%. The overall coulombic efficiency curve of the material is relatively flat and high, indicating that the utilization rate of active materials can always be guaranteed during the charge-discharge cycle.

The redox reversibility can also be studied by the derivative plots (dQ/dV vs. voltage) of the charge-discharge curves. As shown in Figure 4E, there are two reduction peaks at 2.36 and 2.62 V, respectively, corresponding to two step of lithium storage reaction for NDI-NHOH electrode. During the first charge process, two redox peaks occur at 2.43 and 2.67 V, respectively, referring to the reduction reaction. In the following cycles, no deviation in the peak position is observed, however, the peak area is slowly decreased, which means the NDI-NHOH electrode exhibits good reversibility for reduction and oxidation along with slowly capacity fading. These results are well coincided with the work mechanism as displayed in Figure 4A.

Through the charge-discharge curves at 0.05C of Figure 4F, during the charging process, the NDI-NHOH composite cathode displays a bimodal potential curve with a brief plateau at a potential of 2.54 V and a longer plateau at a potential of 3.22 V. While discharging, it shows a symmetrical change in performance between the charge and discharge phases, with a brief plateau at the higher potential of 2.32 V and a lengthy plateau at the lower potential of 2.14 V, respectively. These electrochemical behaviors are consistent with the cyclic voltammetry (CV) characteristics of NDI-NHOH composites. During the first cycle at 0.05C, the composite cathode exhibits a discharge capacity of 77.2 mAh g^−1^, a charge capacity of 83.2 mAh g^−1^, and a decay rate of only 4% after the third cycle. The theoretical capacity and practical capacity at slow rates of NDI-NHOH are higher than those reported for an NDI-based covalent organic framework (TAPB-NDI COF with the theoretical capacity of 63 mAh g^−1^) (Jhulki et al., 2021 ^34^). When it was cycled at 0.1C, a 62.4% of the capacity was still retained after the 20 cycles. The smooth curve shows that the overall discharge capacity trend is similar to that of the first cycle when it is after 20 cycles (Figure 4G). Therefore, this organic cathode shows good long-term cycling stability, proving its potential application prospects in LIBs. However, compared with the CL-DVP-NDI (with the discharge capacity of 121.3 mAh g^−1^ and 84% remaining after 200 cycles) reported by Sharma’s group, the theoretical capacity and cycle performance are lower, which maybe because the NDI polymer can inhibit its dissolution better in organic electrolytes (Sharma et al., 2022 ^35^). But, NDI-NHOH does not require complicated synthesis making its low cost a significant advantage. Moreover, the electrochemical impedance spectra (EIS) of the NDI-NHOH electrode were measured and shown in Figure 4H. The Nyquist plot is constituted of a high-frequency semicircle and a low-frequency straight line. The highest-frequency intercept is attributed to the internal resistance (*R*_*1*_) of total resistance of the electrolyte, separator and current collector (Zheng et al., 2018 ^36^; Ding et al., 2018 ^37^; Zhao et al., 2018 ^38^). The middle-frequency semicircle is due to the charge transfer resistance (*R*_*2*_). The straight line at the low frequency is associated with lithium-ion diffusion in the electrode. The charge-transfer resistance (*R*_*2*_) for NDI-NHOH electrode is 697.1 Ω. Furthermore, the electronic conductivity (*σ*) is calculated from *R*_*2*_ *via* Equation in Figure 4H, where *l* is the thickness of the electrode (∼30 μm), and A is the area of the electrode (1.13 cm^2^). The calculated electronic conductivity of the NDI-NHOH electrode is 3.81 × 10^−7^ S cm^−1^. These results demonstrate that it can further achieve theoretical lithium storage capacity and electrochemical performance under the conditions of simple synthesis, and also validate the previous theoretical calculations and reports of NDI potential by Shi et al. (Shi et al., 2018 ^39^).

## 4 Conclusion

In conclusion, a new electrode material was successfully obtained by extending 2,7-bis(2-((2-hydroxyethyl) amino) ethyl) benzo[lmn] [3,8] phenanthroline-1,3,6,8 (2H, 7H)-tetraonetetrone molecule on NDI through very easy one-step synthesis. And it is prepared as a cathode material by mixing with acetylene black and PVDF binder in NMP. Compared with other reported NDI electrode materials that require high-temperature preparation, condensation and a series of high-cost synthesis methods, the preparation of NDI-NHOH is much shorter and the cost is much lower, which is one of the great advantages of this electrode material (Sharma et al., 2022; Mumyatov et al., 2019 ^40^). The relevant physical properties were characterized by XRD, SEM, EDS, IR and XPS detailedly. As a derivative of NDI, this material exhibits a wide electrochemical window of 1.5–3.5V, and the highest discharge specific capacity can reach more than 80 mAh g^−1^ in LIBs. The physical properties also prove that C-N and C=O groups can be used as active sites for redox reactions to provide higher discharge capacity and energy density. Since NDI-NHOH is an organic material, we speculate that it is possible to use NDI-NHOH as a host material in LIBs. Considering the unique properties of NDI-NHOH, such as low density, synthetic gradients and low price, this research may open up new possibilities for the individualized development of cathode materials. Moreover, the electrode material has a faster capacity decay under high current, which is possible because a small number of organics dissolve to electrolyte during charge and discharge, resulting in rapid capacity decay (Lu et al., 2018)^41^. The carbonyl-based organic electrode materials often do not contain redundant non-redox-active functional groups, while it enables carbonyl compounds to provide higher discharge capacity. Therefore, it may be possible to further increase the capacity of these organic cathode materials by introducing additional redox sites into electrode materials or by creating polymers with layered morphologies. (Cui et al., 2021)^42^.

## Data Availability

The original contributions presented in the study are included in the article/supplementary material, further inquiries can be directed to the corresponding authors.
